# In vitro and in vivo susceptibility to sulfadiazine and pyrimethamine of *Toxoplasma gondii* strains isolated from Brazilian free wild birds

**DOI:** 10.1038/s41598-023-34502-3

**Published:** 2023-05-05

**Authors:** Gabriella de Lima Bessa, Ricardo Wagner de Almeida Vitor, Luana Margarida Sabino Lobo, Wagner Martins Fontes Rêgo, Gabriela Carolina Alves de Souza, Rosálida Estevam Nazar Lopes, Júlia Gatti Ladeia Costa, Erica S. Martins-Duarte

**Affiliations:** 1 Laboratório de Toxoplasmose, Departamento de Parasitologia, Instituto de Ciências Biológicas, Universidade Federal de Minas Gerais, Belo Horizonte, Minas Gerais Brazil; 2 Laboratório de Quimioterapia de Protozoários Egler Chiari, Departamento de Parasitologia, Instituto de Ciências Biológicas, Universidade Federal de Minas Gerais, Av. Antônio Carlos, 6627, Pampulha, Belo Horizonte, Minas Gerais 31270-901 Brazil; 3 Universidade Do Estado de Minas Gerais, Unidade Ibirité, Minas Gerais Brazil

**Keywords:** Parasitology, Antimicrobial resistance

## Abstract

Little is known about the existence of drug-resistant *Toxoplasma gondii* strains and their possible impact on clinic outcomes. To expand our knowledge about the existence of natural variations on drug susceptibility of *T. gondii* strains in Brazil, we evaluated the in vitro and in vivo susceptibility to sulfadiazine (SDZ) and pyrimethamine (PYR) of three atypical strains (Wild2, Wild3, and Wild4) isolated from free-living wild birds. In vitro susceptibility assay showed that the three strains were equally susceptible to SDZ and PYR but variations in the susceptibility were observed to SDZ plus PYR treatment. Variations in the proliferation rates in vitro and spontaneous conversion to bradyzoites were also accessed for all strains. Wild2 showed a lower cystogenesis capacity compared to Wild3 and Wild4. The in vivo analysis showed that while Wild3 was highly susceptible to all SDZ and PYR doses, and their combination, Wild2 and Wild4 showed low susceptibility to the lower doses of SDZ or PYR. Interestingly, Wild2 presented low susceptibility to the higher doses of SDZ, PYR and their combination. Our results suggest that the variability in treatment response by *T. gondii* isolates could possibly be related not only to drug resistance but also to the strain cystogenesis capacity.

## Introduction

In Brazil, the clinical manifestations of toxoplasmosis are more severe than in North America and Europe. This variation is possibly related to differences in the circulating strains of *T. gondii*. Notably, while North America and Europe regions show the prevalence of a clonal population of strains belonging to mainly three genotypes, South America has a high diversity of circulating genotypes of *T. gondii*, and more than 100 have been already described^1,2^. Indeed, in Europe and the USA, most human infections occur by avirulent type II strains but in Brazil, most isolates from human cases are due to strains of virulent or intermediate virulent phenotype^3,4^. In addition, due to the great diversity of genotypes, treatment efficacy may differ for each strain or specific genotype^2^.

The first-choice therapy for the treatment of toxoplasmosis is still the combination of pyrimethamine (PYR) and sulfadiazine (SDZ)^5,6^. Although this therapy is usually effective, failures in the long-term treatment of chorioretinitis, congenital toxoplasmosis, and mainly toxoplasmic encephalitis have been reported^5,7,8^. The lack of response to treatment could be related to pharmacological parameters (drug intolerance, poor adherence, and malabsorption) and/or due to infection with drug-resistant parasites^9,10^.

Although the incidence of drug-resistant strains of *T. gondii* is little known, recent studies carried out with atypical strains in Brazil in animal models have confirmed the existence of Brazilian strains of *T. gondii* resistant to treatment^1^. These studies evaluated isolates from human toxoplasmosis^11,12^, animals meant for human consumption^13^, and domestic animals^14^, leading to the identification of seven atypical strains of *T. gondii* with low susceptibility to SDZ and three to PYR^1^.

The lack of knowledge of the impact of natural resistance of atypical strains of *T. gondii* isolated in South America represents an obstacle in the fight against the parasite. Thus, to expand our knowledge about the incidence of natural variations in drug susceptibility of Brazilian isolates, this work evaluated the in vitro and in vivo susceptibility to SDZ and PYR of three atypical strains of *T. gondii*: TgWildBrMG2 (Wild 2), TgWildBrMG3 (Wild 3), and TgWildBrMG4 (Wild 4) isolated from free-living wild birds rescued in Southeastern Brazil^15^.

## Materials and methods

### Host cell

Normal Human Neonatal Dermal Fibroblast cell cultures (NHDF; Lonza®, kindly donated by Dr. Sheila Nardelli, Fiocruz, Paraná) were maintained in RPMI 1640 medium (Gibco) supplemented with 10% fetal bovine serum (SFB) (Gibco), 4 mM L-glutamine, 100 U/ml of Penicillin, 100 μg/ml of Streptomycin and 25 µg/ml of fungizone (complete RPMI medium) at 37 °C and an atmosphere of 5% CO_2_.

### Isolates Wild2, Wild3 and Wild4 of *T. gondii*

Access to Brazilian genetic heritage approved by SisGen protocols A3F9195 and A90ED70. For in vitro assays, tachyzoites of Wild 2, Wild 3 and Wild 4 strains^15^ were maintained in vitro through serial passages in 25 cm^2^ culture flasks of confluent NHDF in a complete RPMI medium. For in vivo assays, tachyzoites of all strains were intra-peritoneally (i.p.) inoculated in female Swiss mice. The peritoneum of the infected mice was washed five to seven days post-inoculation (DPI), and the obtained tachyzoites were filtered through a 3-μm polycarbonate membrane (Millipore Corporation, Bedford, MA, USA) before in vivo assays. Protocols for animal experimentation were properly revised and approved by the Ethics Committee in Animal Experimentation (CEUA) of the Universidade Federal de Minas Gerais, Brazil (CEUA Protocols: 48/2018 and 318/2022).

### Mice

Six to eight months old outbred female Swiss Webster mice weighing 23-25 g were acquired at the Experimental Animal Center of UFMG and were maintained at the animal facility for infected animals of the Department of Parasitology (UFMG). Mice were supplied with water and food ad libitum and maintained under 12 h light/12 h dark–light cycles. All efforts were made to minimize animal suffering during the study. Euthanasia was performed by an i.p. overdose of ketamine and xylazine, in accordance with the Conselho Nacional de Controle de Experimentação Animal (CONCEA) – Brazil (Resolução Normativa CONCEA no 37/2018), properly revised and approved by the Ethics Committee in Animal Experimentation (CEUA) of the Universidade Federal de Minas Gerais, Brazil (CEUA Protocols: 48/2018 and 318/2022).

### Drugs

For in vitro assays, SDZ and PYR (Sigma-Aldrich®) were dissolved in dimethyl sulfoxide (DMSO, Merck) to give stock solution concentrations so that during antiproliferative experiments the final solvent concentration never exceeded 0.1% and had no effect on the proliferation of intracellular parasites and host cells. For in vivo assays, tablets of 500 g SDZ (Laboratório Catarinense, Brazil) and 25 mg PYR (Daraprim, FQM, Brazil) were ground and dissolved in a water solution of 0.25% carboxymethylcellulose as described^11^.

### Proliferation and antiproliferative assay

Monolayers of NHDF cells in 24-well culture plates containing coverslips were infected with fresh-egressed tachyzoites in a parasite-host cell ratio of 5:1. Tachyzoites were allowed to interact with host cells for 6 h, and then cells were washed twice with medium to remove non-adhered parasites. The proliferation of the strains was evaluated after 18 h and 24 h of interaction. For antiproliferative assays, treatment started after 24 h of infection, and all strains were treated with different concentrations of PYR (0.5, 1.0, and 2.0 µM), SDZ (125, 250, and 500 µM), and the combination of SDZ + PYR (15.6 + 0.0625, 31.25 + 0.125, 62.5 + 0.25, and 125 + 0.5 µM) for 24 h^16,17^. At the end of the experiments, infected cells were washed with phosphate-buffered saline (PBS) pH 7.2, fixed with Bouin, and stained with fast Panoptic kit (Laborclin®, Brazil). Coverslips were mounted onto microscope slides with Entelan® (Merck) and the parasite proliferation was recorded in bright-field optical microscopy^17^. All assays were performed in triplicate. The inhibitory concentration of 50% (IC_50_) of the parasite growth was calculated by fitting the values of proliferation in percentage to a non-linear curve followed by dose–response inhibition analysis through log(inhibitor) vs. normalized response in GraphPad Prism8 software.

### Cystogenesis assay

NHDF cells were infected as described above. Cystogenesis were evaluated for 24, 48, and 72 h post-infection and after treatment with drugs for 24 h and 48 h. For that, infected cells were fixed with 4% freshly prepared formaldehyde and then stained with mouse anti-SAG1 antibody (kindly provided by Dr. Tiago Mineo, Universidade de Uberlândia, Brazil) and the lectin *Dolichos biflorus* (DBA) conjugated to rhodamine (Sigma-Aldrich), as described by Martins-Duarte et al.^18^.

### In vivo* assay*

Female Swiss mice were i.p. infected with 10^4^ tachyzoites of each strain. Groups of 7 (17-day treatment assay) or 10 (10-day treatment assay) mice were assigned according to the treatments with different doses of SDZ (10, 40, 160 mg/kg/day), PYR (3.13, 12.5, and 50 mg/kg/day), and SDZ + PYR (10 + 3.13 mg/kg/day)^12^. Treatment started after 2 days of infection and was given once a day, for 10 or 17 days. Drugs were administered orally by gavage (100 μl). The mice survivals after the end of drug administration were followed up for more 18 days. At the end of the experiment, the number of brain cysts and the production of specific antibodies based on ELISA were analyzed according to the methods of Alves and Vitor^14^. The Untreated infected control (UIC) mice received only 100 μl of 0.25% carboxymethylcellulose solution. The in vivo studies were revised and approved by the Ethics Committee in Animal Experimentation (CEUA) of the Universidade Federal de Minas Gerais, Brazil (CEUA Protocols: 48/2018 and 318/2022). A statistician member of CEUA-UFMG revised the number of animals. All methods were performed in accordance with the relevant guidelines and regulations. All methods are reported in accordance with ARRIVE guidelines.

## Results

### Evaluation of proliferation and cystogenesis of isolates

**Figure 1:**
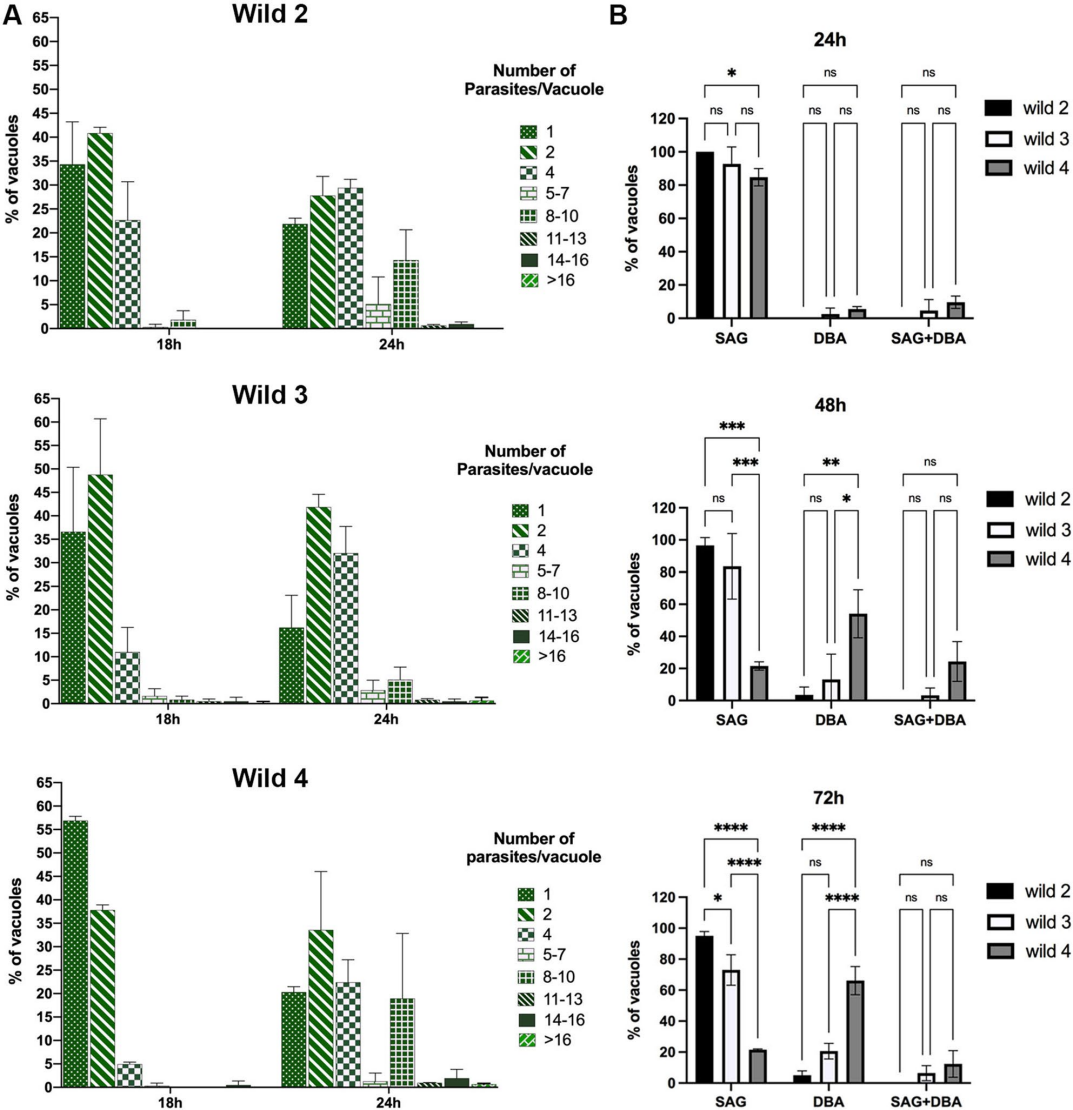
Proliferation and cystogenesis rates of Wild2, Wild3 and Wild4 in vitro. (**A**) Analysis of the proliferation rate in vitro of the three strains after 18 and 24 h of infection. Results represent the mean ± SD of three independent experiments; (**B**) Spontaneous cystogenesis rate in vitro after 24, 48 and 72 h of infection. Parasites were labeled for SAG1 antibody for tachyzoite surface, and DBA-rhodamine for cyst wall. SAG—vacuoles only containing tachyzoites; DBA—vacuoles only containing bradyzoites; SAG + DBA—vacuoles positive for DBA containing SAG positive parasites (intermediate stage). *P < 0.05, **P < 0.01, ***P < 0.001, and ****P < 0.0001 (Two-Way ANOVA Tukey’s multiple comparisons test). Results represent the mean ± SD of two independent experiments.

**Figure 2:**
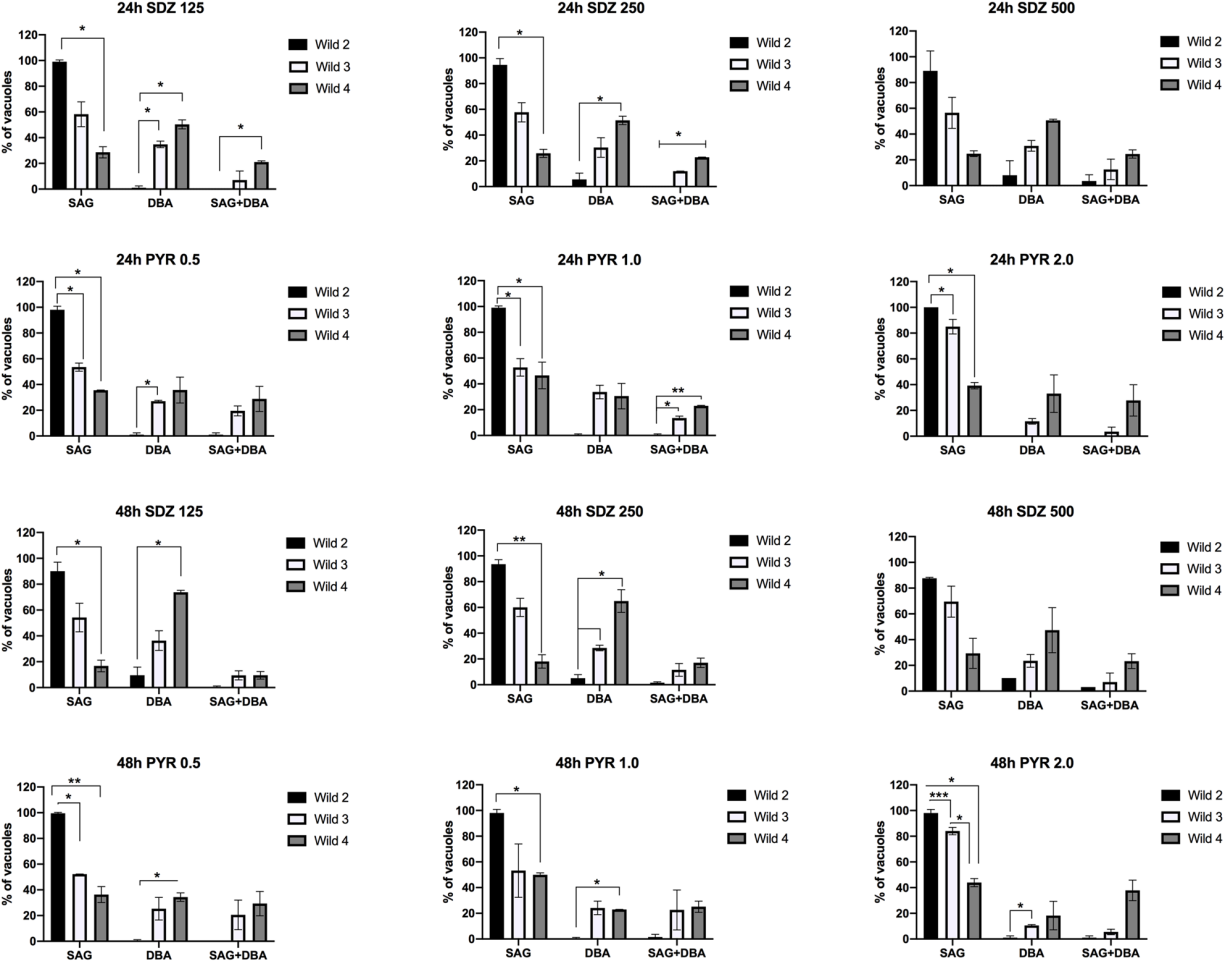
Cystogenesis rate of Wild2, Wild3 and Wild4 in vitro after treatment with SDZ and PYR for 24 and 48 h. Treatment of intracellular parasites were initiated after 24 h of infection and labeled with SAG1 antibody for tachyzoite surface and DBA-rhodamine for cyst wall after 24 and 48 h treatment. SAG—vacuoles only containing tachyzoites; DBA—vacuoles only containing bradyzoites; SAG + DBA—vacuoles positive for DBA containing SAG-positive parasites (intermediate stage). Results represent the mean ± standard deviation of two independent experiments *P < 0.05; **P < 0.01.

The in vitro rates of proliferation of isolates were evaluated for 18 and 24 h post-infection (Fig. 1A). Wild 2 and Wild 4 showed a higher rate of proliferation when compared to Wild3, and after 24 h of infection 14% and 18% of vacuoles presented 8–10 tachyzoites, respectively. At the same time, only 5.1% of the vacuoles of Wild3 had 8–10 tachyzoites (Fig. 1A). However, Wild 2 and Wild 4 showed a remarkable difference in spontaneous cystogenesis in vitro (Fig. 1B). While Wild 2 showed a great majority of vacuoles containing only SAG1-positive parasites, Wild 4 showed a significant amount of spontaneous cystogenesis in vitro, and after 48 h and 72 h of infection 54.1% and 66.1% of vacuoles were, respectively, positives for only DBA (an indicative of cystogenesis). Wild 3 also showed a capacity of spontaneous conversion in vitro, and after 72 h of infection 20% of vacuoles were positive for DBA (Fig. 1B). A higher rate of cystogenesis was also observed when Wild 4 and Wild 3 were treated in vitro with SDZ and PYR (Fig. 2). A significant number of positive vacuoles for only DBA or for both SAG + DBA (intermediate conversion satges) was observed for Wild 3 and Wild 4 after treatment with SDZ and PYR when compared to Wild 2 (Fig. 2).

### Effect of sulfadiazine, pyrimethamine, and their combination on *T. gondii* proliferation in vitro

**Figure 3:**
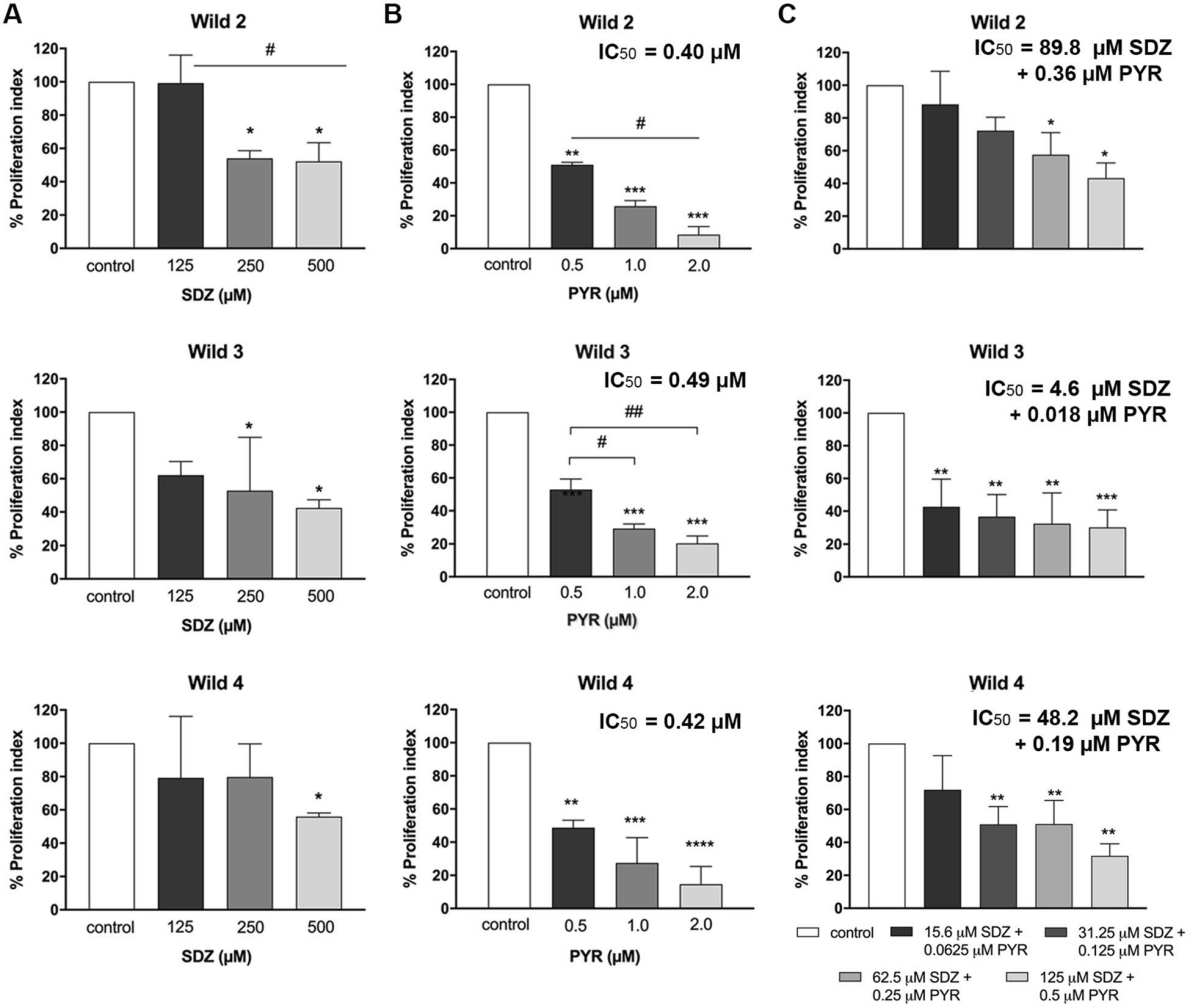
Effect of different concentrations of SDZ, PYR, and SDZ + PYR in the proliferation of Wild2, Wild3, and Wild4 tachyzoites after 24 h of treatment in vitro. (**A**) Effect of SDZ in vitro; (**B**) Effect of PYR in vitro; (**C**) Effect of SDZ + PYR in vitro. *P < 0.05, **P < 0.01, ***P < 0.001, and ****P < 0.0001 in comparison with the untreated group; # and ##*P* < 0.05 comparing to the other experimental groups (One Way ANOVA and Bonferroni post-test). All results represent the mean ± SD of three independent experiments.

Treatment with SDZ resulted in a dose-dependent effect on the proliferation of Wild 2, Wild 3, and Wild 4 strains in vitro. The treatment with 125 µM SDZ did not impact the Wild 2 strain proliferation but concentrations of 250 and 500 µM significantly reduced the proliferation index to 54.0% and 52.2%, respectively (Fig. 3A). The Wild 3 strain was susceptible to all concentrations and showed proliferation indexes of 62.2%, 52.8%, and 52.9% after treatment with 125 μM, 250 μM, and 500 μM of SDZ, respectively (Fig. 3A). Regarding Wild 4 strain, proliferation indexes of 79.2%, 79.7%, and 55.9% were obtained after treatment with 125 μM, 250 μM, and 500 μM of SDZ, respectively (Fig. 3A). The IC_50_s for SDZ were obtained for all the three strains and did not show a significant difference between them (supplemantal Figure S1).

All strains were equally susceptible to all PYR concentrations (Fig. 3B), and IC_50_s of 0.40 µM, 0.49 µM, and 0.42 µM were obtained for Wild2, Wild3, and Wild4, respectively (supplememtal Figure S1). However, a remarkable difference in susceptibility was observed for the combination of SDZ + PYR (Fig. 3C). Wild3 was highly susceptible and a IC_50_ of 4.6 µM SDZ + 0.018 µM PYR was obtained. Wild2 and Wild4 showed a significantly lower susceptibility, and treatment with SDZ + PYR showed IC_50_ of 89.8 µM SDZ + 0.36 µM PYR and 48.2 µM SDZ + 0.19 µM PYR, respectively (supplememtal Figure S1).

### Effect of sulfadiazine, pyrimethamine, and their combination on *T. gondii* proliferation in vivo

**Figure 4:**
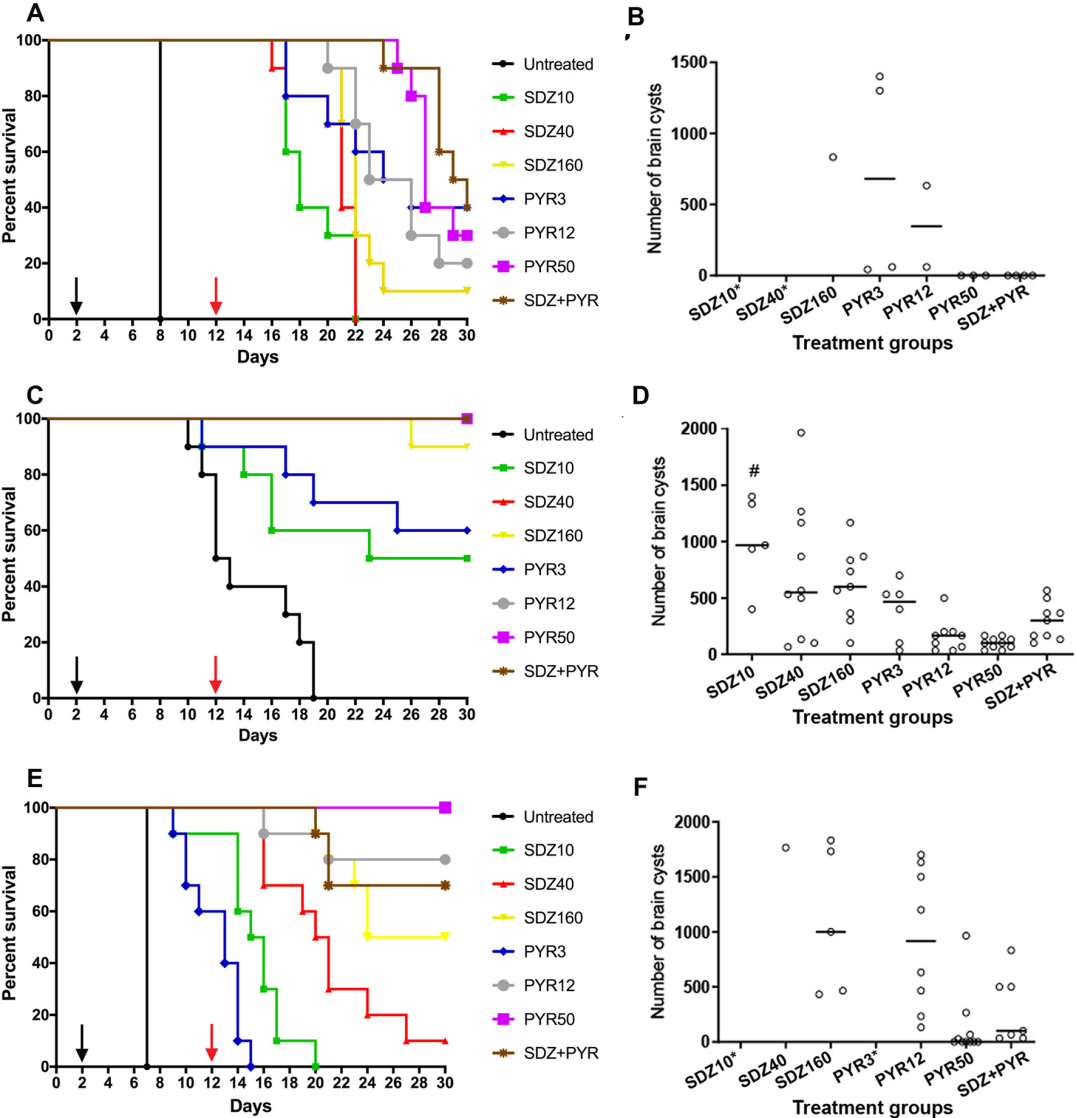
Survival and brain cyst analysis of Swiss mice infected with Wild 2, Wild 3, and Wild 4 after treatment with SDZ, PYR, and their combination for 10 days. (**A**,**C**,**E**) Swiss mice (n = 10 per group) were intraperitoneally infected with 10^4^ tachyzoites of Wild 2 (**A**), Wild 3 (**C**), and Wild 4 (**E**) strains. The oral treatment was initiated on day 2 after infection (black arrows) and lasted for 10 days (day 12; red arrows). Mice survival was followed until day 30. (**B**,**D**,**F**) Brain cyst number quantification in surviving mice after 30 days. #*P* < *0.05* compared to SDZ + PYR (One Way ANOVA and Bonferroni post-test). *No mice survived at those doses.

All strains studied in this work are virulent, and all untreated mice succumbed to death. Wild 2 and Wild 4 infection caused mice death after 8 and 7 days post-infection, respectively and for Wild 3, the last death occurred 19 days post-infection (Fig. 4A,C,E).

In vivo, Wild 2 was the least susceptible to SDZ, all mice that received SDZ10 and SDZ40 died during the experimental period, and the SDZ160 group showed only 10% survival after 30 DPI (Fig. 4A and Table 1). Mice infected with Wild 2 also showed a low survival rate when administered with PYR, and rates of 40, 20 and 30% were observed for the PYR3 (Median of survival = 24 days), PYR12 (Median of survival = 23 days), and PYR50 (Median of survival = 27 days) treatment groups, respectively. The observed survival rate after treatment with SDZ + PYR (40%) was higher than SDZ10 (0%), SDZ40 (0%) and SDZ160 (10%) (Fig. 4A). Serology investigation by ELISA assay showed that except for one animal belonging to the SDZ10 + PYR3 group, surviving mice from all treatment groups were positive for anti-*T. gondii* IgG, proving the success of the experimental infection (data not shown). Brain cysts analysis showed that surviving mice administered with PYR50 or SDZ10 + PYR3 did not have detectable brain cysts, but animals from SDZ160, PYR3, and PYR12 showed brain cysts (Fig. 4B).

**Table 1:** Median of survival and percent of survived mice after the treatments for 10 and 17 days.

Treatment groups	10-days of treatment		17-days of treatment	
	Median of survival (days)	% of survived mice on day 30 DPI	Median of survival (days)	% of survived mice on day 37 DPI
Wild 2				
Untreated	8	0	10	0
SDZ10	18	0	9	0
SDZ40	21	0	25	0
SDZ160	22	10	> 37	85,7
PYR3	25	40	10	0
PYR12	24,5	20	28	14,3
PYR50	27	30	> 37	100
SDZ + PYR	29,5	40	> 37	100
Wild 3				
Untreated	12,5	0	–	–
SDZ10	26,5	50	–	–
SDZ40	> 30	100	–	–
SDZ160	> 30	90	–	–
PYR3	> 30	60	–	–
PYR12	> 30	100	–	–
PYR50	> 30	100	–	–
SDZ + PYR	> 30	100	–	–
Wild 4				
Untreated	7	0	12	0
SDZ10	15,5	0	15	0
SDZ40	20,5	10	22	0
SDZ160	27	50	> 37	85,7
PYR3	13	0	24	14,3
PYR12	> 30	80	> 37	57,1
PYR50	> 30	100	> 37	100
SDZ + PYR	> 30	70	> 37	100

The Wild 3 strain was the most susceptible to SDZ and PYR. The treatment of with SDZ10, SDZ40, or SDZ160 led to mice survival rates of 50%, 100%, and 90%, respectively (Fig. 4C). Similarly, treatment with PYR3 showed 60% survival, and all animals treated with PYR12 or PYR50 survived until the end of the experiment (Fig. 4C and Table 1). All mice administered with SDZ10 + PYR3 also survived (Fig. 4C). All 60 surviving mice showed IgG-antibodies to *T. gondii*, except for one animal belonging to the PYR12 group that presented a negative result (data not shown). All surviving mice infected with Wild 3 strain had brain cysts. There was a trend toward a decrease in the total number of brain cysts associated with increasing dosages of SDZ or PYR. Statistical difference was observed in the number of cysts in mice treated with SDZ10 compared to the SDZ10 + PYR3 combination (Fig. 4D).

Wild 4 were also lowly susceptible to SDZ10 or SDZ40 treatments (0 and 10% of survival, respectively), but 50% of mice treated with SDZ160 survived (Fig. 4E). For the PYR3 (Median of survival = 13 days), PYR12, and PYR50 groups (Median of survival > 30 for both), survival rates were 0%, 80%, and 100%, respectively (Table 1). Although the dosages administered to the SDZ10 and PYR3 groups were non-effective in preventing mortality, the SDZ10 + PYR3 treated group achieved 70% survival. All surviving mice showed positive serology to *T. gondii*, proving the success of the experimental infection (data not shown). Brain cyst number analysis showed that 25 of the 31 surviving mice had brain cysts. However, the differences in brain cyst numbers were not statistically significant between treatment groups (Fig. 4F).

Statistical analysis of brain cyst number showed that a significant difference was obtained for Wild 2-infected mice compared to Wild 3 after treatment with PYR50 and to Wild 3 and Wild 4 after treatment with SDZ10 + PYR3 (Figure S2).

**Figure 5:**
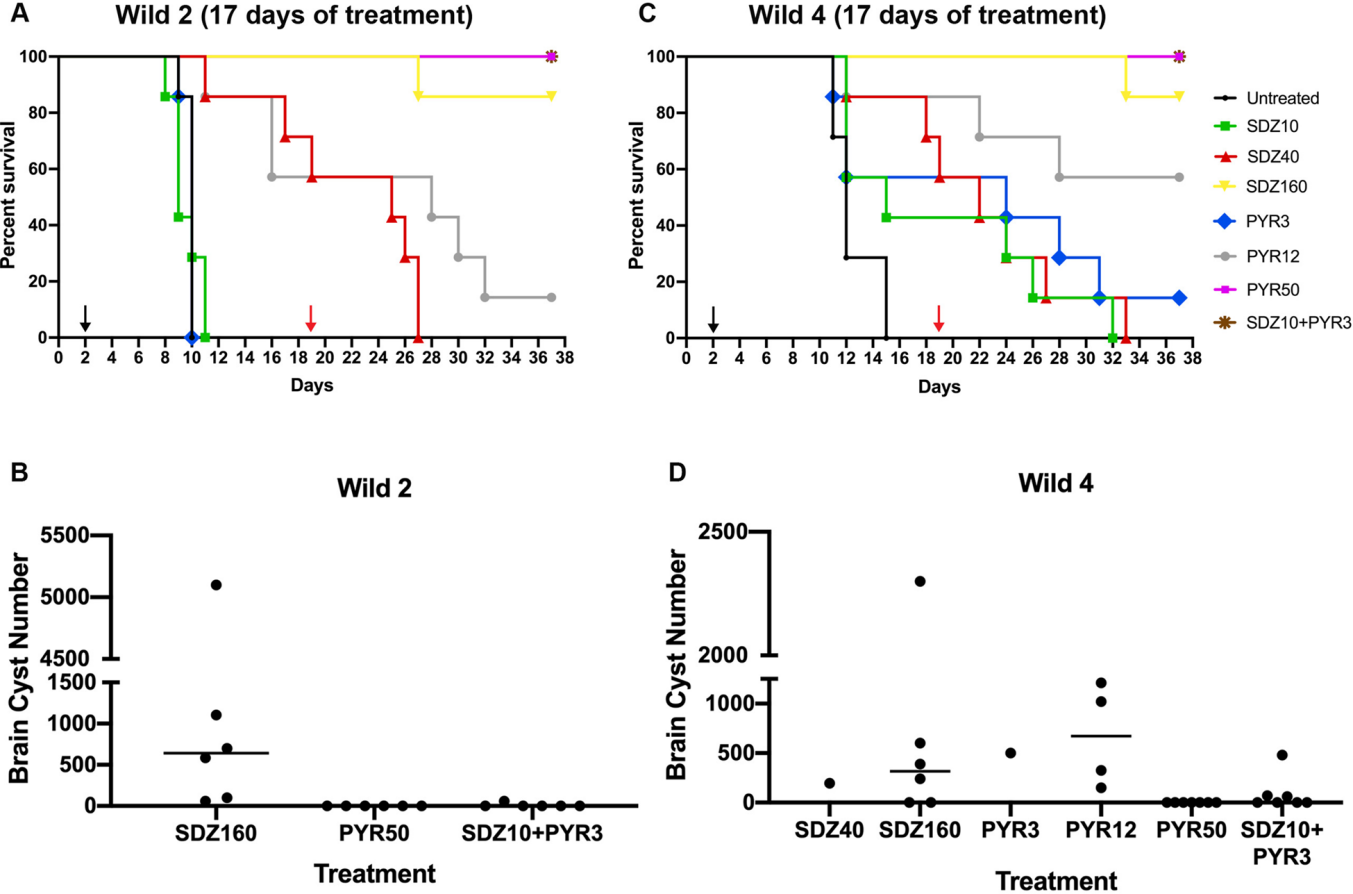
Survival and brain cyst analysis of Swiss mice infected with Wild 2 and Wild 4 after treatment with SDZ, PYR, and their combination for 17 days. (**A**,**C**) Swiss mice (n = 7 per group) were intraperitoneally infected with 10^4^ tachyzoites of Wild2 (**A**) and Wild4 (**C**). The oral treatment was initiated on day 2 (black arrow) and lasted for 17 days (day 19; red arrow). Mice survival was followed until day 37. (**B**,**D**) Brain cyst number quantification in surviving mice after 37 days.

To better investigate the susceptibility of Wild 2 and Wild 4 strains to SDZ and PYR in vivo, additional groups of treatments were performed in which infected mice were treated for more 7 days (10 + 7 days), resulting in 17 days of treatment (Fig. 5). Untreated mice infected with Wild 2 died after 10 days of infection (Fig. 5A). All mice administered with SDZ10 and SDZ40 also died after 10 and 27 days, respectively (Fig. 5A). However mice treated with SDZ160 for 17 days showed rates of higher than animals treated for 10 days (Fig. 4A), and only one mice died at day 27 (survival rate of 85%) (Fig. 5A and Table 1). All mice treated with PYR3 died. However higher survival rates were obtained for treatments with PYR50 (100%) and the combination of SDZ10 + PYR3 (100%) (Fig. 5A and Table 1). Serology investigation showed that all survived animals at the end of experiment were positive for anti-*T. gondii* IgG (data not shown). Brain cysts analysis showed that all survived mice administered with PYR50 and five of SDZ + PYR did not have detectable brain cysts. Survived mice of SDZ160 were positive for brain cysts (Fig. 5B).

Similar results were obtained for mice infected with Wild 4 strain (Fig. 5C). All untreated mice died within 11–15 days of infection. While all mice administered with SDZ10 (Median survival = 15 days) and SDZ40 (Median survival = 22 days) died, SDZ160 treatment led to a mice survival of 85% (Fig. 5C and Table 1). Treatment with PYR3 for 17 days did not enhanced mice survival but increased the median of survival in 9 days. Similar rates and medians of survival were obtained for PYR12, and PYR50 after 17 days compared to 10 days. However a increase in the rate of mice survival was seen with SDZ + PYR after 17-days treatment (Fig. 5C and Table 1). Serology investigation showed that all survived animals at the end of experiment were positive for anti-*T. gondii* IgG (data not shown). Brain cysts analysis showed that all survived mice administered with PYR50 did not have detectable brain cysts. With exception of one mice from SDZ + PYR groups all the other mice were positive for brain cysts (Fig. 5D).

## Discussion

Previous studies reported differences in the susceptibility of atypical strains of *T. gondii* to the SDZ, PYR, and their combination. However, only in vivo models were investigated^11,12^. Here we compared both the in vitro and in vivo susceptibility of three atypical strains isolated from wild birds to these drugs and investigated the proliferation and cystogenic capabilities of all these strains.

In vitro analysis showed that Wild 2, Wild 3, and Wild 4 strains had no remarkable variation in susceptibility to PYR and SDZ (Fig. 3 and Figure S1). Previous works studying the susceptibility in vitro of seventeen *T. gondii* strains from different genotypes, including clonal and atypical, found that PYR IC_50_ varied from 0.28 to 1.57 μM^9^. Variations in IC_50_ of those strains were related to the proliferation capability of each, and those with higher proliferation rates used to show a higher IC_50_, but this had no relation with resistance^9^. Concerning the highly virulent RH strain, studies in vitro showed that this was inhibited by PYR with IC_50_s ranging from 0.23 to 0.9 µM^9,10,17,19,20^. According to the distribution of IC_50_s observed for 16 strains, Meneceur et al.^9^ estimated that less than 0.1% of strains would have an IC_50_ greater than 0.52 mg/L (2.09 µM) for PYR. In human patients with cerebral toxoplasmosis, PYR reaches a serum concentration of 7.6 µM when administrated in a dose of 350 mg/week^21^. Thus, according to the PYR IC_50_ obtained for Wild 2 (0.40 µM), Wild 3 (0.49 µM), and Wild 4 (0.42 µM), we can rule out that these strains are directly resistant to PYR. Concerning SDZ, the Wild 2 strain showed sensitivity only to the highest concentrations (250 and 500 µM), and Wild 4 to 500 µM (Fig. 3). In contrast, the Wild 3 strain showed a tendency of inhibition with 125 µM. Differences in the susceptibility to the different concentrations of SDZ could be explained by the variations in proliferation rates between the three strains. As observed, Wild 2 and Wild 4 have a higher proliferation rate when compared to Wild 3 (Fig. 1A). Previous studies showed an IC_50_ of 260 µM for the RH strain and 176 µM for ME49 after treatment with SDZ for 72 h^19^, but two naturally resistant strains had IC_50_s higher than 3.5 mM. Equally, laboratory-resistant RH and Me49 strains obtained after induction with a gradual increase of SDZ concentrations also resulted in IC_50_ values higher than 3.5 mM^19^. All strains tested in the present study showed a significant reduction in their proliferation when treated with 500 µM of SDZ for 24 h (Fig. 3A) and IC50s similar to those of susceptible strains (Figure S1).

Surprisingly a remarkable difference was seen for in vitro treatment with the combination of SDZ + PYR. Wild 2 and Wild 4 showed IC_50_s 19 and 10 times higher than Wild 3 IC_50_, respectively (Fig. 3C and S1). Unfortunately, other in vitro studies about the susceptibility of *T. gondii* to current drugs did not investigate the effect of this combination. Indeed, this is the first work that investigated the activity of SDZ + PYR in vitro against Brazilian atypical isolates. However, with a similar methodology used in this work, part of this group obtained an IC_50_ of 15 µM SDZ + 0.060 µM PYR, when in combination, after 24 of treatment against the highly virulent RH strain^17^. Thus, the IC_50_s for Wild 2 and Wild 4 after 24 h of treatment are higher than the IC_50_ for RH, which is recognized as susceptible strain to SDZ + PYR. This shows that the higher IC_50_s obtained for Wild 2 and Wild 4 are not related to differences in their proliferation rate once the RH strain shows a cell doubling cycle of 5-7 h^22^, which is higher than the mentioned strains (Fig. 1A).

The mechanisms involved in the variations to susceptibility to SDZ and PYR by *T. gondii* strains are not completely understood. Most of the previous studies did not show a correlation between polymorphisms and/or overexpression of the *dhfr* (dihydrofolate reductase) and *dhps* (Dihydropteroate synthase) genes and the differences in the susceptibility to SDZ and PYR in *T. gondii*. These results demonstrate that the resistance mechanisms in this parasite could be different^9,11,12,19^. Indeed, another study with two resistant strains to SDZ did not show alterations in transporters of family ABC, proteins known to be involved in drug resistance^23^. Using a proteomic approach, Doliwa et al.^23^ observed that 44% of proteins were overexpressed in the resistant strains of *T. gondii*. These results suggest that metabolic alterations, for example, could be involved in the low susceptibility to antifolates by some strains of *T. gondii* and would explain differences seen only for the combination of SDZ + PYR in this study.

Regarding the in vivo susceptibility, Wild 2 and Wild 4 showed a significant difference from Wild 3 for all therapeutic regimens (Fig. 4). Compared to Wild 2 and Wild 4, Wild 3 showed a lower proliferation rate and a higher susceptibility to SDZ and SDZ + PYR in vitro (Figs. 1, 4). Furthermore, although all three strains have an intermediate virulent phenotype in mice, Wild 3 has a different combination of virulence alleles than the other two strains^15^. All three strains share the same alleles for GRA15, ROP5, ROP18, and ROP17 genes, but Wild 2 and Wild 4 strains carry the type I/III allele of ROP16, and the Wild 3 strain carries the type II allele of ROP16^15^. ROP16 is a kinase, and the I/III allele is responsible for directly phosphorylating the transcription factors STAT3 and STAT6, promoting their activation and down-regulation of pro-inflammatory cytokine signaling and the induction of the infected macrophages to an alternatively (M2) activated phenotype, respectively; this would make the strains harboring the type I allele more virulent^24^. As type II ROP16 is a poor activator of STAT3 and STAT6, in infections with strains carrying this allele, macrophages are generally polarized toward the M1 phenotype, which favors the control of parasite proliferation^25,26^. The presence of the type II allele of ROP16 in Wild 3 possibly enhances mice survival during treatment with SDZ and PYR once that immune system and drugs could act together in reducing the parasite burden. Other strains of the same genotype of Wild3 (#11) also showed greater susceptibility to in vivo treatment with SDZ and PYR^12,14^.

However, Wild 2 and Wild 4 carry the same combinations of the virulence factors GRA15, ROP5, ROP16 ROP18, and ROP17 but still showed differences in survival rates after treatment with drugs. Animals infected with Wild 2 showed less susceptibility to treatments with SDZ, PYR, and their combination. In vitro antiproliferative results could explain the difference concerning the treatment with the combination of SDZ + PYR, but not for SDZ or PYR alone. Interestingly, the two strains showed a remarkable difference in spontaneous cystogenesis in vitro (with or without the presence of SDZ and PYR), and Wild 4, even being a virulent strain, had a high rate of conversion compared to Wild 2. During the course of the infection, the conversion to the bradyzoite stage is essential to cease the acute phase of the disease, characterized by the presence of fast-dividing tachyzoites, which causes tissue damage and death^27^. Thus, the cystogenic phenotype of a strain could favor the cessation of the acute phase and mice survival. Interestingly, the extension of treatment from 10 to 17 days increased the survival of mice infected with Wild 2 and administered with SDZ160, PYR50 and SDZ + PYR (Fig. 5 and Table 1). Survived mice from Wild 2 infection mostly presented no detectable brain cysts after treatment with those regimens after 10 or 17-days treatment. Thus, the extension in the number of treatment days would allow the drugs to clarify the remaining tachyzoites from mice tissues infected with Wild 2.

This is the first study that compared drug susceptibility and cystogenesis capacity of *T. gondii* strains, and more studies in this sense are necessary to confirm if there is a correlation between the capacity of cystogenesis and drug susceptibility in vivo. Besides, it is important to point out that there is a scarcity of in vitro studies evaluating the effectiveness of SDZ and PYR in atypical strains of *T. gondii* or even the cystogenic capacity of any strains previously studied for susceptibility. Thus, further studies to evaluate the efficacy of SDZ and PYR, alone or in association in vitro and in vivo, together with the phenotypic characterization of proliferation and encystment capacity in a higher number of *T. gondii* strains of different genotypes are necessary. The enhancement of these data would allow a better understanding of how drug resistance and parasite biology influence the differences in drug susceptibility observed in vivo.

## Supplementary Information

Supplementary Figures.

## Data Availability

The raw data used for the graphs are available upon request from the corresponding author.
