# Supplementary material for: In vitro and in vivo susceptibility to sulfadiazine and pyrimethamine of Toxoplasma gondii strains isolated from Brazilian free wild birds
Source: Sci Rep. 2023 May 5;13:7359. doi: 10.1038/s41598-023-34502-3 (PMC10162961; doi:10.1038/s41598-023-34502-3)
Supplement: Supplementary file 1 — Supplementary Figures. [file 41598_2023_34502_MOESM1_ESM.pdf]

In vitro and _in vivo_ susceptibility to sulfadiazine and pyrimethamine of _Toxoplasma gondii_ strains isolated from Brazilian free wild bird

Gabriella de Lima Bessa[1,#], Ricardo Wagner de Almeida Vitor[1,#], Luana Margarida Sabino Lobo[1], Wagner Martins Fontes Rêgo[1], Gabriela Carolina Alves de Souza[2]; Rosálida Estevam Nazar Lopes[1]; Júlia Gatti Ladeia Costa[1,3]; Erica Santos Martins-Duarte[2*]

Supplementary Figures

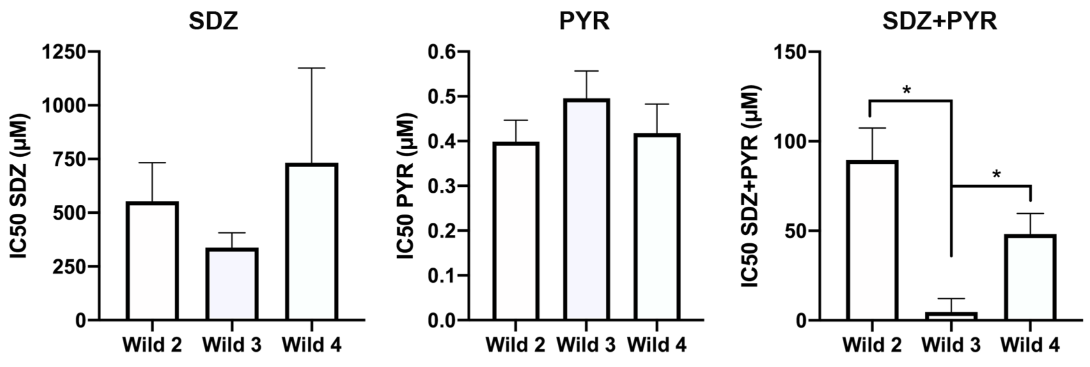

Figure S1. Comparison between IC50 obtained for Wild 2, Wild 3, and Wild 4 after treatment with sulfadiazine (SDZ), pyrimethamine (PYR) and the combination of SDZ + PYR (IC50 is represented by the SDZ value). * $p < 0.05$

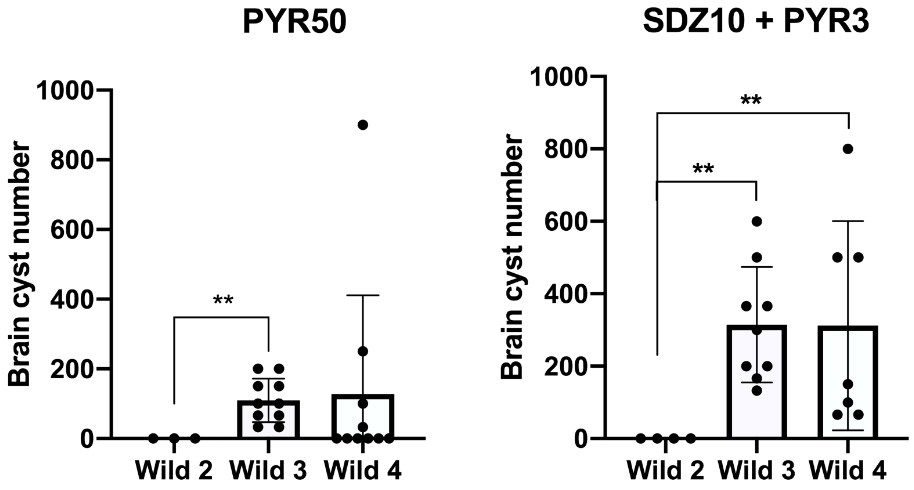

Figure S2. Comparison of the number of brain cysts between mice infected with Wild 2, Wild 3, and Wild 4 after 10 days of treatment with 50 mg/kg/day PYR and 10 mg/kg/day SDZ + 3.13 mg/kg/day PYR. ** $p < 0.001$
